# Poly[[μ-bis­(4-nitro­phen­yl) phosphato-κ^2^ *O*,*O*′]sodium]

**DOI:** 10.1107/S1600536813019260

**Published:** 2013-07-20

**Authors:** Aleksandra Gerus, Tadeusz Lis

**Affiliations:** a University of Wroclaw, Faculty of Chemistry, 14 Joliot-Curie St, 50-383 Wroclaw, Poland

## Abstract

The title compound, [Na(C_12_H_8_N_2_O_8_P)], consists of one Na^+^ cation and one bis­(*p*-nitro­phen­yl)phosphate anion with a considerable distortion of the phosphate tetra­hedron due to the presence of two P—O ester bonds. The anion bridges five Na^+^ cations whereby each cation is chelated by the nitro O atoms of one anion and bonded *via* a nitro O atom and phosphate O atoms to four other anions. This bridging arrangement leads to the formation of double layers parallel to (001). Adjacent layers are linked through weak C—H⋯O hydrogen bonds.

## Related literature

For hydrolytic cleavage of the phospho­diester bond in bis­(*p*-nitro­phen­yl)phosphate (BNPP) and related systems, see: Belousoff *et al.* (2009 ▶); Branum *et al.* (2001 ▶); Chang *et al.* (2009 ▶); Liu *et al.* (2004 ▶); Mancin *et al.* (2005 ▶); Oh *et al.* (1996 ▶); Sredhera & Cowan (2001 ▶). For crystal structures containing the BNPP entity, see: Bazzicalupi *et al.* (2004 ▶); Bond *et al.* (1985 ▶); Fry *et al.* (2003 ▶); Jurek & Martell (1999 ▶); Král *et al.* (2006 ▶); Pletcher *et al.* (1972 ▶); Sax *et al.* (1970 ▶, 1971 ▶); Warden *et al.* (2005 ▶); Yoo *et al.* (1975 ▶).
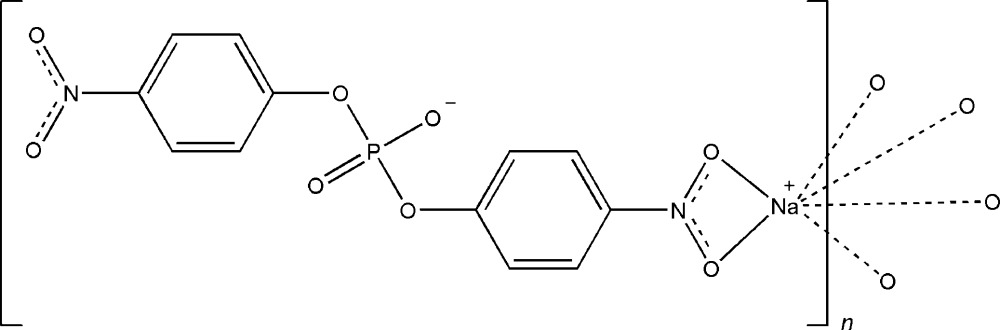


## Experimental

### 

#### Crystal data

[Na(C_12_H_8_N_2_O_8_P)]
*M* *_r_* = 362.16
Triclinic, 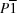

*a* = 6.963 (2) Å
*b* = 9.844 (3) Å
*c* = 11.213 (3) Å
α = 103.93 (3)°
β = 105.83 (3)°
γ = 106.38 (3)°
*V* = 666.1 (3) Å^3^
*Z* = 2
Mo *K*α radiation
μ = 0.29 mm^−1^
*T* = 100 K
0.30 × 0.27 × 0.25 mm

#### Data collection

Agilent Xcalibur (Onyx with CCD camera) diffractometer
Absorption correction: analytical (*CrysAlis PRO*; Agilent, 2011 ▶) *T*_min_ = 0.918, *T*_max_ = 0.931
13418 measured reflections
6748 independent reflections
5542 reflections with *I* > 2σ(*I*)
*R*_int_ = 0.014

#### Refinement

*R*[*F* ^2^ > 2σ(*F* ^2^)] = 0.030
*wR*(*F* ^2^) = 0.090
*S* = 1.07
6748 reflections
217 parameters
H-atom parameters constrained
Δρ_max_ = 0.66 e Å^−3^
Δρ_min_ = −0.33 e Å^−3^

### 

Data collection: *CrysAlis PRO* (Agilent, 2011 ▶); cell refinement: *CrysAlis PRO*; data reduction: *CrysAlis PRO*; program(s) used to solve structure: *SHELXS97* (Sheldrick, 2008 ▶); program(s) used to refine structure: *SHELXL97* (Sheldrick, 2008 ▶); molecular graphics: *XP* in *SHELXTL* (Sheldrick, 2008 ▶); software used to prepare material for publication: *publCIF* (Westrip, 2010 ▶).

## Supplementary Material

Crystal structure: contains datablock(s) I, New_Global_Publ_Block. DOI: 10.1107/S1600536813019260/wm2751sup1.cif

Structure factors: contains datablock(s) I. DOI: 10.1107/S1600536813019260/wm2751Isup2.hkl

Additional supplementary materials:  crystallographic information; 3D view; checkCIF report

## Figures and Tables

**Table:** 

P1—O31	1.4733 (8)
P1—O41	1.4834 (7)
P1—O11	1.6266 (8)
P1—O21	1.6287 (12)
O1—Na	2.9377 (18)
O2—Na	2.4618 (11)
O4—Na^i^	2.3853 (11)
O31—Na^ii^	2.2386 (10)
O41—Na^iii^	2.3135 (9)
O41—Na^iv^	2.4065 (14)

**Table:** 

O31—P1—O41	119.67 (5)
O31—P1—O11	110.86 (4)
O41—P1—O11	110.02 (4)
O31—P1—O21	110.48 (5)
O41—P1—O21	110.21 (4)
O11—P1—O21	92.20 (4)

Symmetry codes: (i) 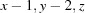; (ii) 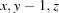; (iii) 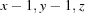; (iv) 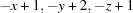.

**Table 2:** Hydrogen-bond geometry (Å, °)

*D*—H⋯*A*	*D*—H	H⋯*A*	*D*⋯*A*	*D*—H⋯*A*
C2—H2⋯O3^v^	0.95	2.50	3.1865 (15)	129
C21—H21⋯O1^vi^	0.95	2.53	3.3337 (18)	142

Symmetry codes: (v) 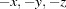; (vi) 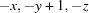.

**Fig. 1.**
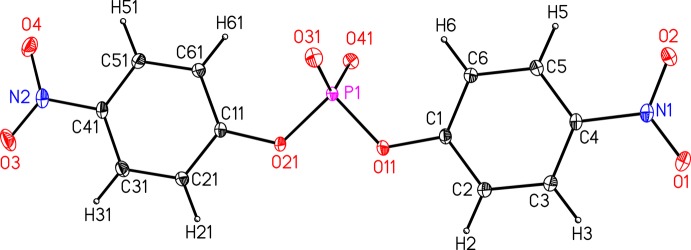
The molecular structure and numbering scheme for the BNPP anion. Displacement ellipsoids are shown at the 50% probability level.

**Fig. 2.**
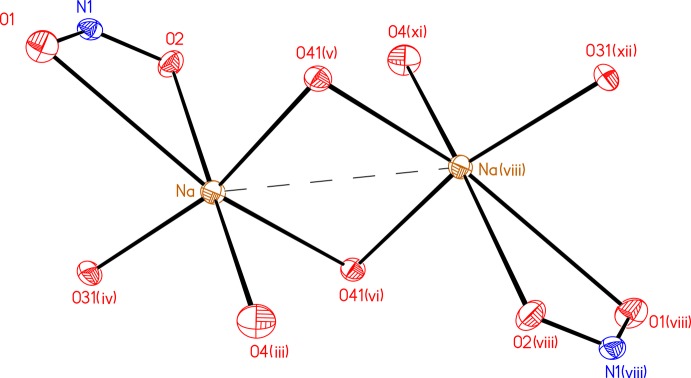
Coordination sphere of the Na+ cation in (I). Displacement ellipsoids are shown at the 50% probability level. [Symmetry codes: (iii) x + 1, y + 2, z; (iv) x, y + 1, z; (v) x + 1, y + 1, z; (vi) -x + 1, -y + 2, -z + 1; (viii) -x + 2, -y + 3, -z + 1; (xi) -x + 1, -y + 1, -z + 1; (xii) -x + 2, -y + 2, -z + 1].

**Fig. 3.**
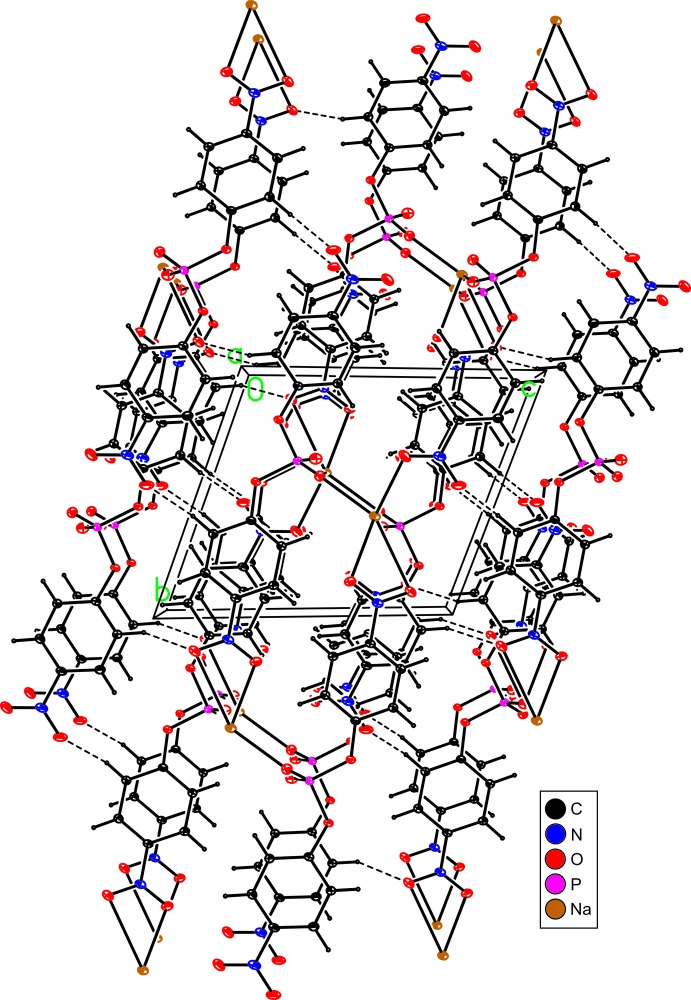
Projection of (I) along [100] showing the double layers. Dashed lines indicate C—H···O hydrogen bonds. Displacement ellipsoids are shown at the 50% probability level.

**Fig. 4.**
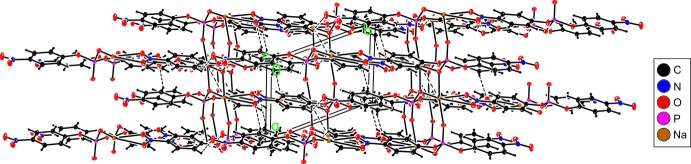
The packing diagram for crystal of the title salt (I). Displacement ellipsoids are shown at 50% probability level.

## supplementary crystallographic information

### Comment

Phosphate diester hydrolysis is a reaction of continuing interest since such process is of fundamental biological importance. Currently, there is much interest in developing artificial nucleases that hydrolyze the phosphate diester bonds in RNA and DNA (Sredhera & Cowan, 2001; Belousoff *et al.*, 2009; Branum *et al.*, 2001). Thus, there have been numerous model studies devoted to understanding how metalloenzymes hydrolyze phosphate diesters (Mancin *et al.*, 2005; Liu *et al.*, 2004). In most cases the substrate of choice is the "activated" phosphate diester bis(*p*-nitrophenyl)phosphate (BNPP). It is considered as an activated phosphate diester because the *p*-nitro groups draw electron density away from the phosphorus atom as well as help to stabilize the negatively charged leaving group (Jurek & Martell, 1999). For this reason the bis(*p*-nitrophenyl)phosphate anion is a popular model substrate for kinetic studies of the hydrolytic cleavage of the phosphodiester bond similar to DNA (Chang *et al.*, 2009; Oh *et al.*, 1996).

BNPP is commercially available as a sodium salt. There are many references concerning solid state studies of BNPP acting as a ligand in complexes or as an anion in salts. The first publications referring to BNPP describe salts of local anesthetics (Sax *et al.*, 1970, 1971; Pletcher *et al.*, 1972; Yoo *et al.*, 1975). The structure of BNPP has been observed also in many macrocyclic complexes (Král *et al.*, 2006; Bazzicalupi *et al.*, 2004; Fry *et al.*, 2003; Warden *et al.*, 2005). Here we report the structure of BNPP as a sodium salt, [Na(C_12_H_8_N_2_O_8_P)], (I).

Compound (I) crystallizes with one bis(*p*-nitrophenyl)phosphate anion (Fig. 1) and one sodium cation in the asymmetric unit. The phenyl rings are almost coplanar. The interplanar angle between two phenyl rings amounts to 2.36 (3)°. The nitro group O1—N1—O2 is rotated 2.00 (4)° from the phenyl ring C1—C6 and the second nitro group O3—N2—O4 is rotated 9.01 (4)° from the phenyl ring C11—C61. The phosphate group is highly distorted from the ideal tetrahedral geometry (Table 1). In the anion there are two shorter P—O bonds of 1.4733 (8) Å and 1.4834 (7) Å, and two longer P—O ester bonds lengths of 1.6266 (8) Å and 1.6287 (12) Å. The shortest bond is P–O31. A little longer than the P–O31 bond is the P–O41 bond, bridging Na^+^ ions. Both O11 and O21 atoms involved in longer ester bonds are attached to aryl rings. The average P—O distance is 1.55 Å, but individual P—O bonds in the structure of compound (I) show how much the phosphate group is deformed. In previous reports containing BNPP anions the most similar deviations for P—O ester bond length in the phosphate group has been observed for these four examples (Sax *et al.*, 1970, 1971; Pletcher *et al.*, 1972; Yoo *et al.*, 1975).

The bond angles in the phosphate group distinctly deviate from the ideal value of 109.5°. The average value for the O–P–O angle is 108.9°, however, individual angles show considerable deviations. The smallest angle is 92.20 (4)° for O11—P1—O21, that is ArO—P—OAr. This deviation can be correlated with the corresponding bond lengths. Such a small angle has not been observed in any previous report of a BNPP structure. The most comparabler value of an O—P—O angle is 95.3 (2)° (Bond *et al.*, 1985). The largest angle is 119.67 (5)° for O31—P—O41, and the four remaining angles are about 110°.

In (I), the coordination geometry of Na^+^ ion is irregular, with an overall coordination number of six [5 + 1] . The Na^+^ ion is coordinated by five symmetry-related BNPP anions *via* oxygen atoms. It is chelated by one anion in a bidentate mode (*via* O1 and O2), and coordinated by four anions in a monodentate manner (*via* O31^iv^, O4^iii^, O41^v^ and O41^vi^) (Fig. 2). The Na—O distances are in the range 2.2386 (10) to 2.9377 (18) Å (Table 2). In the structure there is also a short Na···Na distance of 3.253 (2) Å, and two sodium cations are bridged by two O atoms (denoted as O41 in Fig. 2), forming a dimeric sub-structure with a four-membered ring (Figs. 2 and 4). The cations and anions are arranged in double layers parallel to (001) (Figs. 3 and 4). Adjacent layers are linked through weak C—H···O hydrogen bonds existing between H atoms of the aromatic rings and nitro O atoms (Fig. 3, Table 2).

### Experimental

The bis(*p*-nitrophenyl)phosphate sodium salt was purchased from Sigma-Aldrich. Yellow crystals were obtained after several days by slow evaporation of an aqueous solution.

### Refinement

All H atoms were introduced in geometrically calculated positions, with C—H = 0.95 A ° and *U*_iso_(H) = 1.2Ueq(C).

### Crystal data

**Table:**

[Na(C_12_H_8_N_2_O_8_P)]	*Z* = 2
*M**_r_* = 362.16	*F*(000) = 368
Triclinic, *P*1	*D*_x_ = 1.806 Mg m^−^^3^
Hall symbol: -P 1	Mo *K*α radiation, λ = 0.71073 Å
*a* = 6.963 (2) Å	Cell parameters from 8925 reflections
*b* = 9.844 (3) Å	θ = 3.2–37.5°
*c* = 11.213 (3) Å	µ = 0.29 mm^−^^1^
α = 103.93 (3)°	*T* = 100 K
β = 105.83 (3)°	Block, yellow
γ = 106.38 (3)°	0.30 × 0.27 × 0.25 mm
*V* = 666.1 (3) Å^3^	

### Data collection

**Table:**

Agilent Xcalibur (Onyx with CCD camera) diffractometer	6748 independent reflections
Radiation source: fine-focus sealed tube	5542 reflections with *I* > 2σ(*I*)
Graphite monochromator	*R*_int_ = 0.014
ω and π scans	θ_max_ = 37.6°, θ_min_ = 3.2°
Absorption correction: analytical (*CrysAlis PRO*; Agilent, 2011)	*h* = −11→10
*T*_min_ = 0.918, *T*_max_ = 0.931	*k* = −16→15
13418 measured reflections	*l* = −19→15

### Refinement

**Table:**

Refinement on *F*^2^	Primary atom site location: structure-invariant direct methods
Least-squares matrix: full	Secondary atom site location: difference Fourier map
*R*[*F*^2^ > 2σ(*F*^2^)] = 0.030	Hydrogen site location: inferred from neighbouring sites
*wR*(*F*^2^) = 0.090	H-atom parameters constrained
*S* = 1.07	*w* = 1/[σ^2^(*F*_o_^2^) + (0.0486*P*)^2^ + 0.1337*P*] where *P* = (*F*_o_^2^ + 2*F*_c_^2^)/3
6748 reflections	(Δ/σ)_max_ = 0.001
217 parameters	Δρ_max_ = 0.66 e Å^−^^3^
0 restraints	Δρ_min_ = −0.33 e Å^−^^3^

### Special details

**Table:**

Geometry. All e.s.d.'s (except the e.s.d. in the dihedral angle between two l.s. planes) are estimated using the full covariance matrix. The cell e.s.d.'s are taken into account individually in the estimation of e.s.d.'s in distances, angles and torsion angles; correlations between e.s.d.'s in cell parameters are only used when they are defined by crystal symmetry. An approximate (isotropic) treatment of cell e.s.d.'s is used for estimating e.s.d.'s involving l.s. planes.
Refinement. Refinement of *F*^2^ against ALL reflections. The weighted *R*-factor *wR* and goodness of fit *S* are based on *F*^2^, conventional *R*-factors *R* are based on *F*, with *F* set to zero for negative *F*^2^. The threshold expression of *F*^2^ > σ(*F*^2^) is used only for calculating *R*-factors(gt) *etc*. and is not relevant to the choice of reflections for refinement. *R*-factors based on *F*^2^ are statistically about twice as large as those based on *F*, and *R*- factors based on ALL data will be even larger.

### Fractional atomic coordinates and isotropic or equivalent isotropic displacement parameters (Å^2^)

**Table:**

	*x*	*y*	*z*	*U*_iso_*/*U*_eq_	
P1	0.19163 (3)	0.36887 (2)	0.291283 (19)	0.00783 (4)	
O1	0.52988 (11)	1.11256 (7)	0.18582 (7)	0.01642 (12)	
O2	0.66594 (11)	1.15233 (7)	0.39510 (6)	0.01488 (11)	
O3	−0.28509 (13)	−0.46498 (7)	0.15506 (8)	0.02195 (14)	
O4	−0.14079 (12)	−0.34783 (8)	0.36644 (7)	0.01817 (13)	
O11	0.18884 (10)	0.45097 (6)	0.18143 (6)	0.01210 (10)	
O21	0.02554 (10)	0.20680 (6)	0.18007 (6)	0.01107 (10)	
O31	0.40483 (10)	0.36277 (7)	0.34892 (6)	0.01340 (11)	
O41	0.08681 (9)	0.42769 (7)	0.37940 (6)	0.01131 (10)	
N1	0.55665 (11)	1.06716 (8)	0.27967 (7)	0.01084 (11)	
N2	−0.18533 (12)	−0.34891 (8)	0.25185 (8)	0.01290 (12)	
C1	0.28334 (12)	0.60309 (8)	0.21207 (7)	0.00932 (12)	
C2	0.24915 (13)	0.65553 (9)	0.10570 (8)	0.01132 (12)	
H2	0.1654	0.5862	0.0187	0.014*	
C3	0.33698 (13)	0.80825 (9)	0.12681 (8)	0.01110 (12)	
H3	0.3144	0.8448	0.0551	0.013*	
C4	0.45900 (12)	0.90670 (8)	0.25538 (8)	0.00970 (12)	
C5	0.49639 (13)	0.85587 (9)	0.36171 (8)	0.01053 (12)	
H5	0.5815	0.9255	0.4484	0.013*	
C6	0.40903 (13)	0.70311 (9)	0.34079 (8)	0.01043 (12)	
H6	0.4341	0.6669	0.4126	0.013*	
C11	−0.01420 (12)	0.07325 (8)	0.20306 (8)	0.00914 (11)	
C21	−0.14071 (13)	−0.05705 (9)	0.09256 (8)	0.01090 (12)	
H21	−0.1902	−0.0492	0.0077	0.013*	
C31	−0.19405 (13)	−0.19754 (9)	0.10643 (8)	0.01187 (13)	
H31	−0.2790	−0.2867	0.0320	0.014*	
C41	−0.11984 (13)	−0.20435 (9)	0.23230 (8)	0.01057 (12)	
C51	0.00777 (13)	−0.07667 (9)	0.34263 (8)	0.01163 (13)	
H51	0.0579	−0.0854	0.4271	0.014*	
C61	0.06168 (13)	0.06395 (9)	0.32862 (8)	0.01120 (12)	
H61	0.1489	0.1526	0.4033	0.013*	
Na	0.75570 (5)	1.41177 (4)	0.39243 (3)	0.01082 (7)	

### Atomic displacement parameters (Å^2^)

**Table:**

	*U*^11^	*U*^22^	*U*^33^	*U*^12^	*U*^13^	*U*^23^
P1	0.00772 (8)	0.00661 (8)	0.00894 (8)	0.00226 (6)	0.00311 (6)	0.00274 (6)
O1	0.0212 (3)	0.0124 (3)	0.0172 (3)	0.0056 (2)	0.0065 (2)	0.0091 (2)
O2	0.0167 (3)	0.0091 (2)	0.0144 (3)	0.0027 (2)	0.0032 (2)	0.0020 (2)
O3	0.0321 (4)	0.0079 (3)	0.0231 (3)	0.0030 (3)	0.0129 (3)	0.0025 (2)
O4	0.0225 (3)	0.0154 (3)	0.0206 (3)	0.0075 (2)	0.0087 (3)	0.0116 (2)
O11	0.0172 (3)	0.0066 (2)	0.0100 (2)	0.00113 (19)	0.0046 (2)	0.00292 (18)
O21	0.0137 (2)	0.0062 (2)	0.0103 (2)	0.00136 (19)	0.00201 (19)	0.00335 (18)
O31	0.0084 (2)	0.0132 (3)	0.0176 (3)	0.0045 (2)	0.0038 (2)	0.0042 (2)
O41	0.0108 (2)	0.0121 (2)	0.0121 (2)	0.00466 (19)	0.00572 (19)	0.00352 (19)
N1	0.0107 (3)	0.0084 (3)	0.0147 (3)	0.0041 (2)	0.0052 (2)	0.0049 (2)
N2	0.0142 (3)	0.0093 (3)	0.0190 (3)	0.0054 (2)	0.0094 (2)	0.0063 (2)
C1	0.0102 (3)	0.0068 (3)	0.0107 (3)	0.0024 (2)	0.0041 (2)	0.0031 (2)
C2	0.0127 (3)	0.0092 (3)	0.0100 (3)	0.0020 (2)	0.0031 (2)	0.0035 (2)
C3	0.0123 (3)	0.0092 (3)	0.0111 (3)	0.0031 (2)	0.0036 (2)	0.0044 (2)
C4	0.0101 (3)	0.0072 (3)	0.0122 (3)	0.0031 (2)	0.0044 (2)	0.0038 (2)
C5	0.0119 (3)	0.0078 (3)	0.0106 (3)	0.0031 (2)	0.0035 (2)	0.0025 (2)
C6	0.0123 (3)	0.0086 (3)	0.0097 (3)	0.0031 (2)	0.0037 (2)	0.0035 (2)
C11	0.0094 (3)	0.0073 (3)	0.0110 (3)	0.0029 (2)	0.0039 (2)	0.0036 (2)
C21	0.0126 (3)	0.0084 (3)	0.0101 (3)	0.0025 (2)	0.0036 (2)	0.0028 (2)
C31	0.0136 (3)	0.0082 (3)	0.0124 (3)	0.0025 (2)	0.0054 (3)	0.0024 (2)
C41	0.0121 (3)	0.0074 (3)	0.0142 (3)	0.0040 (2)	0.0068 (2)	0.0046 (2)
C51	0.0130 (3)	0.0104 (3)	0.0118 (3)	0.0040 (2)	0.0040 (2)	0.0053 (2)
C61	0.0130 (3)	0.0086 (3)	0.0099 (3)	0.0024 (2)	0.0027 (2)	0.0031 (2)
Na	0.00911 (14)	0.00915 (14)	0.01433 (15)	0.00301 (11)	0.00447 (12)	0.00451 (12)

### Geometric parameters (Å, º)

**Table:**

P1—O31	1.4733 (8)	C2—C3	1.3861 (12)
P1—O41	1.4834 (7)	C2—H2	0.9500
P1—O11	1.6266 (8)	C3—C4	1.3908 (14)
P1—O21	1.6287 (12)	C3—H3	0.9500
O1—N1	1.2282 (10)	C4—C5	1.3895 (12)
O1—Na	2.9377 (18)	C5—C6	1.3867 (12)
O2—N1	1.2399 (12)	C5—H5	0.9500
O2—Na	2.4618 (11)	C6—H6	0.9500
O3—N2	1.2288 (12)	C11—C61	1.3976 (12)
O4—N2	1.2335 (11)	C11—C21	1.3995 (14)
O4—Na^i^	2.3853 (11)	C21—C31	1.3857 (12)
O11—C1	1.3663 (11)	C21—H21	0.9500
O21—C11	1.3668 (10)	C31—C41	1.3894 (12)
O31—Na^ii^	2.2386 (10)	C31—H31	0.9500
O41—Na^iii^	2.3135 (9)	C41—C51	1.3862 (14)
O41—Na^iv^	2.4065 (14)	C51—C61	1.3877 (12)
N1—C4	1.4552 (12)	C51—H51	0.9500
N2—C41	1.4560 (12)	C61—H61	0.9500
C1—C2	1.3987 (12)	Na—Na^v^	3.253 (2)
C1—C6	1.3998 (14)		
			
O31—P1—O41	119.67 (5)	C1—C6—H6	120.6
O31—P1—O11	110.86 (4)	O21—C11—C61	123.21 (8)
O41—P1—O11	110.02 (4)	O21—C11—C21	116.04 (7)
O31—P1—O21	110.48 (5)	C61—C11—C21	120.74 (8)
O41—P1—O21	110.21 (4)	C31—C21—C11	120.28 (8)
O11—P1—O21	92.20 (4)	C31—C21—H21	119.9
N1—O1—Na	83.76 (6)	C11—C21—H21	119.9
N1—O2—Na	106.72 (6)	C21—C31—C41	118.15 (8)
N2—O4—Na^i^	116.10 (7)	C21—C31—H31	120.9
C1—O11—P1	123.69 (6)	C41—C31—H31	120.9
C11—O21—P1	123.67 (6)	C51—C41—C31	122.37 (8)
P1—O31—Na^ii^	157.93 (4)	C51—C41—N2	117.95 (8)
P1—O41—Na^iii^	143.20 (5)	C31—C41—N2	119.61 (8)
P1—O41—Na^iv^	129.49 (4)	C41—C51—C61	119.45 (8)
Na^iii^—O41—Na^iv^	87.11 (4)	C41—C51—H51	120.3
O1—N1—O2	122.74 (7)	C61—C51—H51	120.3
O1—N1—C4	119.12 (8)	C51—C61—C11	119.01 (8)
O2—N1—C4	118.13 (8)	C51—C61—H61	120.5
O1—N1—Na	72.71 (6)	C11—C61—H61	120.5
O2—N1—Na	50.43 (5)	O31^vi^—Na—O41^vii^	165.08 (3)
C4—N1—Na	167.00 (5)	O31^vi^—Na—O4^viii^	99.55 (4)
O3—N2—O4	123.14 (8)	O41^vii^—Na—O4^viii^	81.55 (4)
O3—N2—C41	119.25 (8)	O31^vi^—Na—O41^iv^	101.98 (5)
O4—N2—C41	117.59 (8)	O41^vii^—Na—O41^iv^	92.89 (4)
O11—C1—C2	115.91 (8)	O4^viii^—Na—O41^iv^	80.28 (4)
O11—C1—C6	123.34 (8)	O31^vi^—Na—O2	84.38 (4)
C2—C1—C6	120.75 (7)	O41^vii^—Na—O2	92.94 (4)
C3—C2—C1	120.25 (8)	O4^viii^—Na—O2	172.26 (3)
C3—C2—H2	119.9	O41^iv^—Na—O2	105.55 (4)
C1—C2—H2	119.9	O31^vi^—Na—O1	74.29 (5)
C2—C3—C4	118.44 (8)	O41^vii^—Na—O1	93.25 (4)
C2—C3—H3	120.8	O4^viii^—Na—O1	128.23 (3)
C4—C3—H3	120.8	O41^iv^—Na—O1	151.44 (3)
C5—C4—C3	121.88 (8)	O2—Na—O1	46.28 (3)
C5—C4—N1	118.65 (8)	O31^vi^—Na—N1	76.99 (4)
C3—C4—N1	119.43 (8)	O41^vii^—Na—N1	94.92 (4)
C6—C5—C4	119.77 (8)	O4^viii^—Na—N1	151.70 (3)
C6—C5—H5	120.1	O41^iv^—Na—N1	128.02 (4)
C4—C5—H5	120.1	O2—Na—N1	22.85 (2)
C5—C6—C1	118.90 (8)	O1—Na—N1	23.53 (2)
C5—C6—H6	120.6		
			
O31—P1—O11—C1	−74.91 (8)	C4—C5—C6—C1	−0.33 (12)
O41—P1—O11—C1	59.73 (8)	O11—C1—C6—C5	−179.42 (7)
O21—P1—O11—C1	172.18 (6)	C2—C1—C6—C5	1.12 (12)
O31—P1—O21—C11	53.96 (7)	P1—O21—C11—C61	8.66 (11)
O41—P1—O21—C11	−80.51 (7)	P1—O21—C11—C21	−172.40 (6)
O11—P1—O21—C11	167.21 (6)	O21—C11—C21—C31	−178.45 (7)
P1—O11—C1—C2	−174.63 (6)	C61—C11—C21—C31	0.52 (12)
P1—O11—C1—C6	5.90 (11)	C11—C21—C31—C41	0.45 (12)
O11—C1—C2—C3	179.50 (7)	C21—C31—C41—C51	−1.26 (12)
C6—C1—C2—C3	−1.01 (12)	C21—C31—C41—N2	175.67 (7)
C1—C2—C3—C4	0.09 (12)	O3—N2—C41—C51	−174.72 (8)
C2—C3—C4—C5	0.72 (12)	O4—N2—C41—C51	6.77 (11)
C2—C3—C4—N1	178.70 (7)	O3—N2—C41—C31	8.22 (12)
O1—N1—C4—C5	177.82 (7)	O4—N2—C41—C31	−170.30 (8)
O2—N1—C4—C5	−1.43 (11)	C31—C41—C51—C61	1.08 (12)
O1—N1—C4—C3	−0.23 (11)	N2—C41—C51—C61	−175.90 (7)
O2—N1—C4—C3	−179.48 (7)	C41—C51—C61—C11	−0.07 (12)
C3—C4—C5—C6	−0.60 (12)	O21—C11—C61—C51	178.18 (7)
N1—C4—C5—C6	−178.60 (7)	C21—C11—C61—C51	−0.71 (12)

Symmetry codes: (i) *x*−1, *y*−2, *z*; (ii) *x*, *y*−1, *z*; (iii) *x*−1, *y*−1, *z*; (iv) −*x*+1, −*y*+2, −*z*+1; (v) −*x*+2, −*y*+3, −*z*+1; (vi) *x*, *y*+1, *z*; (vii) *x*+1, *y*+1, *z*; (viii) *x*+1, *y*+2, *z*.

### Hydrogen-bond geometry (Å, º)

**Table:**

*D*—H···*A*	*D*—H	H···*A*	*D*···*A*	*D*—H···*A*
C2—H2···O3^ix^	0.95	2.50	3.1865 (15)	129
C21—H21···O1^x^	0.95	2.53	3.3337 (18)	142

Symmetry codes: (ix) −*x*, −*y*, −*z*; (x) −*x*, −*y*+1, −*z*.
